# Views and experiences of nurse practitioners and medical practitioners with collaborative practice in primary health care – an integrative review

**DOI:** 10.1186/1471-2296-14-132

**Published:** 2013-09-05

**Authors:** Verena Schadewaldt, Elizabeth McInnes, Janet E Hiller, Anne Gardner

**Affiliations:** 1 Faculty of Health Sciences, Australian Catholic University, Melbourne, Australia; 2 Nursing Research Institute, Australian Catholic University and St Vincents and Mater Health, St Vincent’s Hospital, Sydney, Australia; 3 Faculty of Health Sciences, Australian Catholic University, Canberra, Australia

**Keywords:** Collaboration, Cooperative behaviour, Interprofessional relations, Attitude of health personnel, Nurse practitioners, Primary health care

## Abstract

**Background:**

This integrative review synthesises research studies that have investigated the perceptions of nurse practitioners and medical practitioners working in primary health care. The aggregation of evidence on barriers and facilitators to working collaboratively and experiences about the processes of collaboration is of value to understand success factors and factors that impede collaborative working relationships.

**Methods:**

An integrative review, which used systematic review processes, was undertaken to summarise qualitative and quantitative studies published between 1990 and 2012. Databases searched were the Cochrane Library, the Joanna Briggs Institute Library, PubMed, Medline, CINAHL, Informit and ProQuest. Studies that met the inclusion criteria were assessed for quality. Study findings were extracted relating to a) barriers and facilitators to collaborative working and b) views and experiences about the process of collaboration. The findings were narratively synthesised, supported by tabulation.

**Results:**

27 studies conducted in seven different countries met the inclusion criteria. Content analysis identified a number of barriers and facilitators of collaboration between nurse practitioners and medical practitioners. By means of data comparison five themes were developed in relation to perceptions and understanding of collaboration. Nurse practitioners and medical practitioners have differing views on the essentials of collaboration and on supervision and autonomous nurse practitioner practice. Medical practitioners who have a working experience with NPs express more positive attitudes towards collaboration. Both professional groups report concerns and negative experiences with collaborative practice but also value certain advantages of collaboration.

**Conclusions:**

The review shows that working in collaboration is a slow progression. Exposure to working together helps to overcome professional hurdles, dispel concerns and provide clarity around roles and the meaning of collaboration of NPs and MPs. Guidelines on liability and better funding strategies are necessary to facilitate collaborative practice whether barriers lie in individual behaviours or in broader policies.

## Background

A nurse practitioner (NP) in primary health care collaborates on average with 4.4 medical practitioners (MPs) and most of these MPs work on-site with the NP [1]. In most countries with NPs, it is a legal requirement for NPs to have a formally established collaborative agreement for MP support or supervision [2-4]. The legal obligation to collaborate with a MP is crucial for NPs to enable full practice authority and reimbursement of NP services [5,6]. While there is debate about the necessity of this legislative requirement [6,7], it has been identified that a good collaborative relationship can improve patient outcomes such as reduced waiting times, improved prescribing processes, shorter treatment periods and lower costs [8-12]. Furthermore, collaboration increases work satisfaction [13] and decreases the perception of job strain [14] for NPs. The above reasons emphasise the importance of a successful collaborative practice model for MPs and NPs.

Collaboration, as described in the literature, involves trust, mutual respect, shared decision-making and equality [15,16]. Collaboration in practice often does not necessarily include these attributes but rather exists solely through referrals and occasional consultations between health professionals [1,17-19]. A survey of 378 primary health care NPs identified that many bi-directional referrals occur between NPs and family MPs or MPs working in community health centres, but only one-way referrals from NPs to specialists were observed [18]. It appears that collaboration can range from an intense relationship and regular knowledge exchange between NPs and MPs to a more distant and superficial co-existence of services provided by NPs and MPs [19].

No matter what form of collaboration is in place, a number of factors can influence the functioning or failure of collaborative practice between NPs and MPs. Literature reviews [20-26] and primary research [27-31] have highlighted a number of barriers and facilitators to collaborative practice and perceptions of health professionals of working in collaboration. These relate to funding issues, traditional role allocation, legislation, personal experience with and attitudes towards collaboration and organisational aspects [32]. The existing reviews focus on collaboration in multidisciplinary teams, in hospital settings and collaboration between general nurses and MPs. Collaboration between NPs and MPs in primary health care may differ to other settings and roles, because NPs bring increased autonomy to the clinical setting that may challenge the traditionally MP dominated domain of primary health care, where nurses have long been working to support the MP and perform delegated tasks [24,33].

Therefore, this literature review aims at summarising the existing evidence about the views and experiences of NPs and MPs with collaborative practice in primary health care settings. The findings of the review will provide information about health professionals’ understanding of collaboration, the perceived barriers and facilitators to collaborative practice and their attitude about working in collaboration. Since this review aims to aggregate data of qualitative and quantitative evidence and not to re-interpret findings, an integrative synthesis was the method chosen for this literature review [34]. The steps for integrative reviews outlined in Whittemore and Knafl [35] were followed and thematic synthesis for “views studies” applied as described by Harden and Thomas et al. [36,37].

## Methods

A number of methods are available for the synthesis of qualitative and quantitative evidence [35,38-42]. A majority of these methods focus on effectiveness or intervention reviews and add findings of non-experimental research to the synthesis of trials in a separate step (parallel or multi-level synthesis). For this review Whittemore and Knafl’s [35] approach to the synthesis of qualitative and quantitative evidence was chosen because their focus is not on effectiveness reviews and statistical pooling of data. They suggest an integrated approach that is reflected in the simultaneous process of synthesising data from quantitative and qualitative research under themes that were addressed in studies using a variety of designs and methods. However, Whittemore and Knafl [35] lack a detailed description of how data extraction, the analysis and synthesis can be undertaken; therefore, we relied on other researchers’ methods to guide these processes. We drew on principles described by the Joanna Briggs Institute [43], the Cochrane Qualitative and Implementation Methods Group [38] and the thematic synthesis approach for qualitative data developed by Thomas and Harden [37] for literature reviews on participant views. The latter matched the purpose of this review that also looked at views and perceptions.

### Eligibility criteria

Studies were included in the review if they focused on a population of NPs (nurses with a postgraduate certification and an advanced level of practice autonomy [44,45]) and MPs in primary health care settings. The outcomes of included studies needed to report on a) facilitators and/or barriers to collaboration and b) experiences and perceptions of NPs and MPs of collaboration. Study designs that generated qualitative or quantitative data were included. Opinion papers and anecdotal reports were excluded.

### Information sources and search strategy

The following databases were searched: Cochrane Library, Joanna Briggs Institute Library of Systematic Reviews, PubMed/MEDLINE, CINAHL, ProQuest (Dissertation and theses) and Informit (Health collection). The review also contains grey literature such as theses and dissertations.

When available medical subject headings or index terms were used in each database. An example of a typical search is shown in the Additional file 1 for the MEDLINE database using OvidSP. The inclusion period of papers comprised the years from January 1990 to September 2012 to ensure the inclusion of papers that reported collaboration between NPs and MPs from countries where the NP role has been implemented for a much longer time and collaboration may be at a more advanced stage than in other countries [46]. No language restrictions were applied.

Results from all databases were combined in Endnote®, duplicates deleted and the results screened by title and abstract for suitability for the literature review. One reviewer examined the full text of potentially relevant papers for final inclusion or exclusion in the review. Reference lists of included papers were screened for eligible studies.

### Assessment of methodological quality

A separate appraisal tool was used for each included study type [35]. The following were chosen due to their brevity, clarity, appropriateness; and because their items covered the most common assessment criteria of other tools:

•For cross-sectional studies – 11 Questions to help you make sense of descriptive/cross-sectional studies [47]

•For surveys – CEBMA Appraisal Questions for a Survey [48]

•For qualitative studies – JBI Qualitative Assessment Research Instrument (QARI) [43]

•For mixed methods research – Scoring System for appraising mixed methods research [49]

No articles were excluded from the review based on their methodological quality to not exclude valuable insights from weaker studies [50], unless findings were not supported by the presentation of appropriate quotations from participants [43].

### Data extraction

Firstly, study details such as the methodology, the population and the context of the study were extracted from each study and organised in an evidence table (Additional file 2: Evidence table). Secondly, findings were extracted from the primary sources into a spreadsheet and grouped under one of the outcome categories: barriers, facilitators, and perceptions/views of collaboration [35]. Findings to be extracted from qualitative studies for the purpose of this review were themes, key concepts or results and conclusions developed by the authors of the papers [37,51]. No direct quotations of individuals were extracted since they were considered raw data and not the outcome of an interpretative process undertaken by the authors [52].

A separate table was created for relevant quantitative data and organised under the same outcome categories as the qualitative data.

### Data analysis and synthesis

Repeated screening of the articles and reading of extracted data in spreadsheets enhanced the iterative process of developing sub-categories [53]. These sub-categories were further collapsed into descriptive themes [37].

As “counting highlights the recognition of patterns in the data” ([54], p.152), a simple listing of the most common statements relating to barriers or facilitators to collaboration was part of the data synthesis. This approach is similar to content analysis, suggested by Dixon-Woods et al. [34] as one possible approach to synthesising results.

Results from quantitative studies were juxtaposed with qualitative findings within each descriptive theme and outlined in a descriptive summary, supported by tabulation of data [55]. Since the synthesis of findings in this review was a meta-aggregation [43] of results, it was summative and did not include the re-interpretation of the primary data [55,56].

## Results

**Figure 1:**
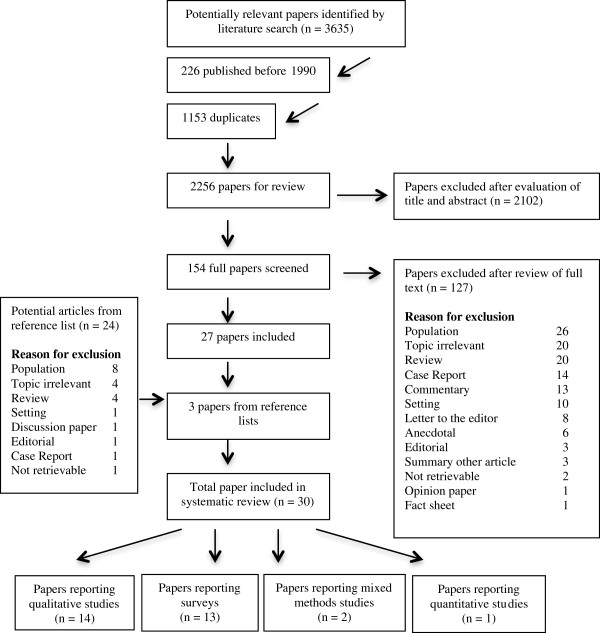
Study selection process.

The literature search identified 3635 papers. After excluding duplicates and papers published before 1990 there were 2256 papers for review. The flow chart in Figure 1 summarises the review process. In total there were 30 papers included in the review, reporting 27 studies. The most common reasons for exclusion were a population other than NPs and MPs in a primary health care setting, no information relevant to the research question or the papers were literature reviews.

There was an almost equal number of papers reporting qualitative studies (n = 14) and surveys (n = 13), whereas there were only two mixed methods study papers and one paper reporting data from a cross-sectional design as part of one of the mixed methods studies. However, most of the surveys applied a mixed-methods design, using open-ended and closed questions. A meta-analysis of quantitative results was not possible because only one study investigated effects of an intervention on perceived collaboration.

The evidence of this review is based on studies including a total of 1641 MPs and 380 NPs (among those were 4 APNs with a similar level of authority than NPs). The majority of studies were undertaken in the US (11) followed by Canada and the UK (6 each) with one study undertaken in each of the Netherlands, Sweden, Ireland and New Zealand.

### Methodological quality of studies

Overall, studies were of moderate quality with some information difficult to assess due to weaknesses in reporting (Additional file 3: Quality appraisal). Issues for qualitative studies were the lack of reporting of a philosophy and the researchers’ background. One study [57] was excluded from the analysis, because no illustrative quotations from participants were provided to assess the credibility of findings [43].

All survey papers reported a clear aim of the study and used the appropriate design to answer the research question. The survey studies lacked sufficient response rates and representativeness of the sample. A major flaw in most studies was the use of self-developed questionnaires without the reporting of their psychometric properties.

Two studies applied a mixed methods design [19,58]. Both studies had clear qualitative objectives and used appropriate qualitative methods for the research process. Both studies did not state the researchers’ background. For the quantitative part, both studies did not apply appropriate sampling procedures and used a convenience sample of one [58] or four [19] practices.

From Way et al.’s comprehensive mixed methods study [19,59,60], one part was published with results from a cross-sectional analysis of referral patterns between NPs and MPs [59]. The use of encounter forms for referral patterns may not be a valid measure for collaboration since it relies on self-report. The strengths and weaknesses of each study are documented in the evidence table (Additional file 2: Evidence table).

### Results – facilitators and barriers of collaboration

Factors facilitating or impeding collaborative practice between NPs and MPs were identified in 18 of the 30 papers, including qualitative, survey and mixed methods studies. Often facilitators were identified as the opposite of obstacles to collaborative practice. Therefore the facilitator and the corresponding barrier were matched and counted as one thematic factor impacting on collaboration. Those factors are listed in order of their frequency of appearance in Table 1.

**Table 1:** Barriers and facilitators to collaboration

**Factors impacting on collaboration**	Frequency*
Clarity of NP role & scope of practice	15
NPs take over workload from MPs	11
Confidence in each other’s competence	11
Complementary skills and practice ideology	9
Knowing the NP/MP & good working relationship	9
Reciprocity (including the absence of hierarchy & control)	9
Clear legal liability	8
Effective communication (including the use of technologies)	8
Financial support for NP role	7
Mutual trust & respect	7
Support from MPs	6
Shared responsibility	6
High level of NP autonomy	5
Working in close physical proximity	4
Regular meetings & time to collaborate	3
Positive attitude towards collaboration	3
Official recognition of NP role	3
Collaboration develops and improves over time	2
MPs’ concern of becoming deskilled (barrier only)	1
MPs feel threatened by NPs (barrier only)	1

*Data were extracted from qualitative, survey and mixed-methods studies. The frequency refers to the number of times each barrier and facilitator was found in 18 studies.

The most common barrier to collaboration was the lack of awareness by MPs of the scope of practice of NPs, their level of education and what is inherent to their role [19,58,60-66]. Collaboration worked well where MPs noted that NPs took over some parts of their workload such as education and follow up care [60], ‘routine cases’ [67] or patients with minor illnesses and chronic diseases [63], so that MPs were able to focus on more complex cases [17]. However, not all MPs have experienced a decrease in workload because NPs would consult the MP for their patients [64] and supervision of NPs increased the workload of MPs [68].

To make collaboration work, NPs and MPs have to be confident in the competence of the collaborating partner. Both professions valued having competent colleagues. For MPs and NPs themselves this also included that NPs were competent in realising their limits and seeking assistance when needed [17,63,69]. While having complementary skills and similar goals was seen as an asset to collaboration [61,70,71], ideological differences in the practice style could cause difficulties in establishing a collaborative relationship [19,60,64,70].

An important factor for successful collaboration was previous experience of working with the NP or MP [19,58,60,63,64,66,70] and having a good relationship [67,70]. Developing a good collaborative relationship took time and improved once the NPs and MPs got to know each other, which also helped to establish trust among the health professionals [63,70,71]. A period of 3–6 months was observed to be sufficient to establish a collaborative relationship [58,63,70].

While the reciprocity of referrals and consultations [19,60,63] as well as the absence of hierarchical structures were considered to foster collaboration, NPs and MPs also reported control issues as a barrier to collaborative practice. NPs often perceived a hierarchical relationship with the MP that was described as a power struggle for NPs [72] and experienced by NPs when the MP decided over the range of tasks to be undertaken by the NP [67]. Medical practitioners reported losing control about patient triage through the introduction of NPs [60].

The fourth common obstacle to work in collaborative practice with a NP was the concern of MPs about legal responsibility. Most considered themselves liable for the care provided by the NP [19,58,60,61,63-65]. An equal amount of findings identified effective communication [70,71,73] as crucial to collaboration. In addition to face-to-face communication, two studies identified the use of technologies such as messaging systems as beneficial for regular communication [19,58].

Nurse practitioners and MPs strongly perceived that economic constraints had a negative impact on collaborative practice. The lack of financial support for the NP role often made employment of a NP not financially viable for a practice setting. There was a perception that the health care system did not sufficiently reimburse NP services [19,61,66,70]. As important as funding for collaborative practice models were trust and respect between NPs and MPs. Mutual trust and respect was perceived by NPs when MPs were referring patients to them [63] or advice seeking was reciprocal [60].

The frequency count of barriers and facilitators to collaboration showed that support from the MPs was crucial to establish a collaborative practice with the NP [61,69]. Other experiences reported by NPs and MPs as important for collaboration were sharing responsibilities of complex cases [61,73] rather than leaving complex cases to either the NP or the MP [61,63,67]. In terms of responsibilities, some MPs perceived that NPs were not prepared to take on the level of responsibility appropriate to the NP role [64]. In general, a high level of NP autonomy was a crucial component to collaboration, because limitations in the NP’s autonomy; in particular their inability to prescribe or order diagnostic tests was found to increase the MPs workload and consequently negatively influence collaborative practice [61,65,69,72].

Further fostering factors were working in close physical proximity or on the same site [19,60,70], taking time for regular meetings [58,70], a positive attitude towards collaboration [70,71]; and the official recognition of the NP role, including the legal protection of the professional title ‘nurse practitioner’ [63,67].

Two quantitative studies investigated what NPs and MPs experienced as barriers or facilitators to collaborative practice and their results support the qualitative findings. In De Guzman et al.’s [13] survey of 29 NPs working at Canadian PHC sites, the NPs stated the unwillingness of specialists to accept their referrals (53.5%), the MPs’ lack of understanding of the NP role (42.8%) and the personality of the MPs (35.7%) as the most common challenges in their collaborative practice with the MPs. Of a list of facilitators of collaboration, NPs identified the trust shown by MPs in making shared decisions (57.1%), the respect shown by the MPs (42.8%) and the personality of the MPs (46.4%) as the most common facilitators [13].

Way et al. [59] considered the imbalance of referrals between NPs and MPs as a barrier to collaborative care because it would indicate a lack of shared care. They found that only 2% of 173 patient encounters with a GP resulted in a referral to a NP in contrast to 16% of 79 patients who saw a NP and were then referred to a MP for follow-up [59].

### Results - experiences and views of collaboration

Qualitative and quantitative studies have identified differences in the perception and understanding of collaboration between NPs and MP. Five descriptive themes were developed from the extracted data, not all of them were found in both qualitative and quantitative data.

#### The essence of collaboration and practice reality

While NPs and MPs agreed on some essential components of collaboration, there were differences in their understanding about several of these components (Table 2).

**Table 2:** Comparison of nurse practitioner and medical practitioner views

**Dimensions of comparison**	**Nurse practitioner views**	**Commonalities**	**Medical practitioner views**
*Important elements of collaboration*	Respect as a health professional,	Working together	Complementary practice style
	Reciprocal relationship	Consultations	Similar vision
		Trust & mutual respect	Shared goals
		Communication	
		Competence	
		Coordination	
		NP autonomy	
		Personality	
		Shared philosophy	
		Sharing	
*Sharing*	Exchange of knowledge and ideas about patient management	Important for collaboration	Shared offices, shared patients
*Working together*	Reciprocal discussion	Important for collaboration	Providing advice to NPs
*Practice reality*	Collaboration can be hierarchical and one-sided; only initiated by NPs for consultation	Perceived level of communication is high Perceived level of collaboration is collegial	Collaboration can be an interdependent and a hierarchical relationship
*Competence*	Defined by MP, pressure to demonstrate competence	Important for collaboration	Important that NP recognises limits
*Autonomy*	NP is autonomous health professional	Important for collaboration	NP is assistant, limited autonomy of NPs
	NP has full responsibility for patient care, consultations with MP when required		NP is autonomous when no MP consultation is required
*Supervision*	Some NPs valued MP input, others felt controlled through supervision	MP is available on site for NP	MPs prefer that NP practices under MP supervision for complex cases

Data extracted from 13 studies.

Two studies explicitly investigated the elements that were important to NPs and MPs about collaboration: working together, consultations, trust and mutual respect, communication, competence, coordination, NP autonomy, the health professionals’ personality and a shared philosophy [61,71]. However, in Hallas et al.’s [71] survey of 24 paediatric NPs and their 24 collaborating paediatricians, NPs understood the term “sharing” as the exchange of ideas and knowledge while MPs referred to shared patients or shared offices. This study also reported that NPs saw collaboration as a reciprocal discussion about patients while MPs described collaboration as advice seeking of NPs.

Characteristics considered essential for MPs but that were not found in NP statements were complementary practice styles and a similar vision [71] or a shared goal [60]. For NPs it is particularly important to be respected as a health professional [71] and to work in a reciprocal relationship [60]. However, in practice, NP-MP work arrangements were often one-sided and lacked reciprocity, with collaboration predominantly initiated by NPs who consulted the MP when a problem was outside their scope of practice [17,19,59]. Since MPs served as a (supervisory) resource for NPs, NPs perceived that they worked in a hierarchical relationship where demonstrating competence was a one-way process [19,70]. NPs stated their experience of being under constant pressure to demonstrate their competence because NP competence was defined by the MPs [60,67].

Three author groups explicitly concluded that collaboration in practice did not reach the ideal [17,58,60] with NPs expecting a collegial relationship with MPs but actually experiencing a more hierarchical situation. While some MPs agreed that collaboration can exist as true reciprocity they rather acknowledged that forms of collaboration range from an interdependent to hierarchical relationship [60]. Contrary to some of these findings, NPs and MPs rated their working relationships with each other as collegial [68] and their level of collaboration and communication as high [74] when measured on attitude scales.

#### Supervision and autonomous practice

The concept of supervision and autonomous NP practice were common themes relating to collaboration. Medical practitioners rarely saw NPs as autonomous health professionals, however attitudes differed between MPs employing a NP and those who did not.

Some MPs saw the NP in the role of an assistant or MP extender [68,70]. Medical practitioners preferred to see the NP practicing under their direct supervision if managing complex cases [68]. The survey of Hallas et al. [71] revealed that some NPs saw supervision as negative, as being controlled by MPs, others valued supervision as having the MP available on site. Similarly, MPs understood supervision as providing consultations to the NPs or simply being available on site. Autonomous NP practice for the NPs comprised full responsibility for patient care with MP consultation when required. In contrast, MPs considered NPs as autonomous when they had no need to consult with a MP [71].

Quantitative data supported these perceptions of supervision and autonomous NP practice. NPs perceived, more than MPs, that they could perform tasks autonomously [62,75]. Some MPs stated that NPs require regular MP supervision [62] and that NPs care for patients who are too complex for the NPs’ skills and knowledge [68]. GPs who worked with a NP were more supportive of NPs performing most tasks without supervision than GPs who worked not with a NP [76].

#### Differences in the views of medical practitioners with and without experience of collaborating with nurse practitioners

Three cross-sectional surveys reported that MPs with previous experience of working with a NP exhibit a more positive attitude towards collaboration with NPs [76-78]. Medical practitioners who had experience in collaborating with a NP were significantly more likely to disagree that NPs provide low-quality primary health care, and more likely to support NP prescribing, consider that NPs can attract new patients, agree that patients accept NPs and believe that NPs free up MP time [77,78]. In Carr et al.’s survey 100% of the GPs who worked with a NP agreed that NP should work in primary health care compared to 89% of the GPs who did not [76]. No qualitative studies investigated those differences.

#### Medical practitioners’ concerns and ambivalence about working with nurse practitioners

Qualitative data revealed a number of concerns of MPs to working in collaboration with NPs. Some of these concerns were also identified as barriers to collaborative practice such as concern about: NP education and competence [66,79], NPs’ limited scope of practice for patients with multiple comorbidities [68], ultimate liability for NP care [79] and financial disadvantages [66]. Other issues for MPs were that they could be left with complex patient cases that increased their workload but also deskilled them in areas taken over by the NP [66]. In Katz & MacDonald’s [79] focus group study of Canadian MPs who had not worked with NPs before, the MPs expressed concern about quality and fragmentation of care. Some MPs stated that they considered the difference of education between NPs and MPs as a barrier to acceptance of NPs as equal partners [79]. In a sample of British GPs, Wilson et al. [66] identified that MPs felt threatened in their role by NPs and were concerned about their professional status and a loss of self-esteem. Furthermore, they stated that a NP would be more expensive to employ than a practice nurse [66].

The ambivalence of MPs was often based in insecurity about the advantages and disadvantages of collaborating with a NP. Marsden & Street [65] found that MPs valued the benefits for patients of longer consultations with the NP but simultaneously were concerned about the cost effectiveness of those consultations. In a study by Dutch researchers [73], MPs stated that prescribing authority for NPs would be more practical for their collaborative practice but they were hesitant to grant their collaborating NP this right. Medical practitioners valued NP competence, however, competence was often equated to the competence of NPs to refer patients outside the NP scope of practice and appropriate consultation with the MPs [19,63,69].

#### Medical practitioners’ reasons for working with nurse practitioners

Medical practitioners who worked in collaboration with a NP, reported that NP tasks may be complementary to the MP’s scope of practice [79] and this was valued by some MPs because they could focus on patients with more complex issues [63]. Nurse practitioners were acknowledged as an extra resource for the MPs [69,79] and one MP perceived the NP as a colleague to discuss patients, specifically their psychosocial needs [65]. Medical practitioners in particular valued NPs’ educational and interpersonal skills [17,65,68].

Three survey studies from the UK [76], US [80] and New Zealand [81] identified that the majority of MPs would be willing either to work in collaboration with or to employ a NP for reasons of increased patient choice, reduced workload, more cost-effective use of resources, MP shortage and reduced waiting times for patients [76].

## Discussion

This review describes the experiences and views of NPs and MPs working collaboratively in primary health care. Summarising quantitative and qualitative data has shown that NPs and MPs rated their collaborative practice experience as collegial [68,74] but at the same time obstacles, concerns and different perceptions were voiced in qualitative inquiries. Nurse practitioners and MPs face a number of barriers when working in collaboration. Concurrently they have found ways to overcome these obstacles and improve the collaborative relationship through negotiation, clarifying roles and creatively working around organisational impediments. Thus, collaboration includes working around barriers and using facilitators for long-term establishment of collaborative practice.

While there was overlap in the majority of components that NPs and MPs considered as essential for collaboration, the detailed analysis revealed that the professions might ascribe a different meaning to these components. This was also the result of a study that investigated collaboration in nursing homes, where advanced practice nurses and MPs used the same terms to define collaboration but had a different understanding about these terms [82].

A fine line lies between MP supervision being perceived as hierarchical or consultative. This perception seemed very much influenced by the individual situation and personality of the health professional. The strong movement seen in the US towards unsupervised NP practice may not be welcomed by all NPs who may find having some medical support reassuring [3,6,83]. However, NPs may wish to work in an autonomous manner and still be able to consult with a medical colleague when needed, identified as one way of collaboration by studies included in this review [17,71]. A survey of primary health care NPs in the US confirmed that NPs provide 80% of their services autonomously or with minimal consultation [1].

Nurse practitioners, more than MPs, seemed confident in autonomous NP practice, but MPs who worked with NPs showed more trust in the NPs’ capabilities and support for autonomous NP work than MPs who lacked this experience [76-78]. The reasons for this may be that the MPs’ work experience with the NP increased their confidence in the benefits of collaboration or that MPs who have a positive attitude about collaboration with a NP are more likely to work with one. Consequently NPs rely on the support and willingness of MPs to work with them. There is evidence from a replication study undertaken in the US that NP-MP collaboration increased since the original survey 20 years earlier [30].

The majority of MPs who had worked with NPs acknowledged that NPs were an asset to the practice and the patients. However, this was limited to tasks undertaken with routine patients. Medical practitioners also valued NP competence, which for some meant NPs who were competent to realise their boundaries and seek advice when appropriate. This reveals a paternalistic attitude of MPs instead of recognising the capabilities of NPs in terms of their professional scope of practice. Finlayson and Raymont [33] raise the point that NP employment through MPs will influence their collaborative relationship because the employer-employee relationship is hierarchical by definition.

Working towards successful collaboration may be achieved through interventions that target effective collaborative practice [19,59,84]. Some of the concerns raised by MPs may be reduced through better information strategies about the NP role and early exposure to interprofessional education [85-88]. The simple use of DVDs explaining the education pathway and the skills of NPs increased significantly the knowledge of primary health care MPs and their positive attitude towards NPs and collaborative practice [89].

### Limitations

No secondary reviewer assisted in the appraisal of studies and extraction of data. The data to be extracted had been specified in advance with the outcome categories and since there has been no re-interpretation of data, it is unlikely that results have been distorted from those of the primary data.

No attempt was made to contact authors, so that the methodological quality may rather relate to reporting quality and the way the study was conducted may be of better quality than reflected in the article. The assessment of qualitative studies was difficult due to the lack of reporting on the researcher’s background. While word limitations may restrain authors from reporting additional information, two sentences about their background and influence on the project would provide the reader with information crucial to establishing the credibility of findings [90].

While all included studies investigated nurse practitioners who were educated at a postgraduate degree level and who practiced at an advanced level that included the diagnosing of patients, regulations around the NP role, licensure and practice vary among and within countries [45,46,91]. Therefore, themes and factors identified in this review may only apply to the particular NP role in the primary health care setting of the country of study.

## Conclusion

This integrative review of literature is important to highlight NPs and MPs experience and perceptions of working collaboratively in primary health care. It is the first review to specifically look at nurse practitioners, not general nurses and to only include studies undertaken in primary health care settings and not secondary or tertiary institutions.

By integrating quantitative and qualitative data a comprehensive synthesis of research evidence on collaboration between NPs and MPs in the primary health care setting was possible. The results of this review show that collaboration develops step by step, that professional hurdles need to be overcome, and that positive experiences of working collaboratively may be the strongest force to promote and advance collaboration between NPs and MPs. Further research into the most effective strategies to prepare NPs and MPs for collaborative practice is necessary. In addition clear policies on liability and funding strategies are necessary to dispel MPs’ concerns and facilitate collaborative practice.

## Abbreviations

NP: Nurse practitioner; MP: Medical practitioner; GP: General practitioner; PHC: Primary health care; UK: United Kingdom; US: United States of America.

## Competing interests

No conflict of interest has been declared by the authors.

## Authors’ contributions

VS study selection, appraisal and analysis, review design, manuscript writing, EM study appraisal, review design, content discussion and manuscript review, JEH and AG content discussion and manuscript review. All authors read and approved the final manuscript.

## Authors’ information

VS: PhD candidate, MHSc, RN, EM: MPH PhD; Associate Professor & Deputy Director, Nursing Research Institute, Australian Catholic University and St Vincents and Mater Health, Sydney. JEH: PhD MPH FPHAA; Associate Dean of Health Sciences (Research) & Professor of Public Health, Australian Catholic University; Adjunct Professor, University of Adelaide. AG: PhD MPH RN; Professor of Nursing, Australian Catholic University; Adjunct Professor, James Cook University.

## Pre-publication history

The pre-publication history for this paper can be accessed here:

http://www.biomedcentral.com/1471-2296/14/132/prepub

## Supplementary Material

Additional file 1 Medline search, Table with Medline search strategy. Click here for file

Additional file 2 Evidence table, Table that provides details of all included studies. Click here for file

Additional file 3 Quality appraisal, Table that outlines the quality appraisal of included studies. Click here for file
