# Supplementary material for: Views and experiences of nurse practitioners and medical practitioners with collaborative practice in primary health care – an integrative review
Source: BMC Fam Pract. 2013 Sep 5;14:132. doi: 10.1186/1471-2296-14-132 (PMC3846155; doi:10.1186/1471-2296-14-132)
Supplement: Additional file 1 — Medline search, Table with Medline search strategy. [file 1471-2296-14-132-S1.pdf]

Medline search strategy

1. *Cooperative Behavior/
2. *Partnership Practice/
3. *Physician-Nurse Relations/
4. *Interprofessional Relations/
5. *Nurse Practitioners/
6. *"Attitude of Health Personnel"/
7. "collaborative practice".ab,ti.
8. collaboration.ab,ti.
9. "nurse practitioner?".ab,ti.
10. 1 or 2 or 3 or 4 or 7 or 8
11. 5 or 9
12. 10 and 11
13. 6 and 11
14. 12 or 13
