# Supplementary material for: Views and experiences of nurse practitioners and medical practitioners with collaborative practice in primary health care – an integrative review
Source: BMC Fam Pract. 2013 Sep 5;14:132. doi: 10.1186/1471-2296-14-132 (PMC3846155; doi:10.1186/1471-2296-14-132)
Supplement: Additional file 2 — Evidence table, Table that provides details of all included studies. [file 1471-2296-14-132-S2.pdf]

Evidence table

Reference	Aim	Methodologies/ Design	Population, sample size, sampling	Context/ Setting	Theoretical frameworks/ data collection methods	Outcomes	Strengths and limitations
**Qualitative studies**							
[61]	To explore and define the meaning of collaborative practice as experienced by the NP and MP.	Exploratory and descriptive qualitative study (Grounded Theory)	3 NP-MP dyads in private practice<br><br>Purposeful sampling	GP practices, South-Eastern US	- Semi-structured individual interviews<br>- Interviews with each NP-MP dyad<br>- Observations<br>- Content analysis	- Developed themes (frequency in data): Personality, Competence, Communication, Autonomy, coordination, Trust, Benefits of collaboration, Barriers<br>- Barriers: Economical, traditional hierarchy, lack of collegial support, lack of autonomy, knowledge deficit, lack of shared responsibility<br>- Some examples of successful collaboration	Limitations: Small sample size; author claims to use grounded theory for data analysis but applies summative content analysis throughout; researcher influence on data not discussed; author states that observations were undertaken but these data do not occur in the data analysis or results; no statement on ethics approval
[60]	To understand the experiences of NPs and MPs working in collaborative practice and to examine the impact of an educational intervention on interprofessional practice (comparison of practices)	Exploratory qualitative study, using narrative analysis, a form of interpretive analysis<br><br>Part of a larger mixed methods study [19,59]	5 NPs and 13 family MPs<br><br>Purposeful sampling	4 rural primary care practices, Ontario, Canada	- Interviews (based on 'Collaboration and Satisfaction About Care Decisions' instrument	- Themes: NPs' scope of practice and NP competence with an emphasis on role clarity and trust; issues around control at the work place; ideological differences regarding disease prevention and health promotion, differences in perceptions about the operation of collaborative practice and understanding that collaborative relationships evolve.<br>- MPs participating in intervention to enhance collaboration indicated afterwards that they still 'rarely' consulted with NPs in their clinic.<br>- The theoretical ideal of collaboration has not been achieved in practice<br>- NP services were underutilized<br>- Referral practices were not reciprocal.<br>- Facilitators: length of time together, proximity to one another, past positive experiences	Strengths: Well described data analysis method; credible representation of participants<br><br>Limitations: Data from 2000; limited generalisability; researcher influence on data not stated
[72]	To explore the current role of advanced NPs in PHC, and how NPs within three different nursing disciplines in PHC developed their roles	Exploratory qualitative study	18 advanced NPs (11 practice) managers)<br><br>Purposive sampling	PHC practices and community centres; West Midlands, UK	- Semi-structured interviews<br>- Content analysis and thematic analysis	- Barriers: NPs felt not supported by MPs, power struggle for NPs as 'handmaiden', lack of understanding of NP role by MPs; limited NP autonomy in regards to prescriptions increases MP workload.<br>- Facilitators: NPs felt supported by MPs, MPs consulted the NPs if they were confident about the NPs' competence.	Strengths: Large sample size; well presented and credible results<br><br>Limitations: Researcher influence on data not stated; research philosophy not stated
[73]	To explore the value of NPs and to describe their role in PHC	Mixed-methods long-term study (4 years)<br><br>This paper reports qualitative results	7 NPs<br>7 GPs<br>(= 7 groups)<br><br>Convenience sample	PHC practices, Netherlands	- 29 interviews<br>- observations from consultations (quant data)<br>- job satisfaction questionnaire	- 5/7 MPs considered NP's communication skills as good.<br>- 4/7 NPs were very satisfied with MP supervision.<br>- 6/6 MPs were very satisfied with the NP as PHC professional<br>- NPs and MPs share care of patients with complex needs<br>- MPs are mentors for NPs<br>- Role clarity is important<br>- MP noted that NP consultations differ to their own	Strengths: Participant voices are well presented<br><br>Limitations: Authors lack to link qualitative findings to results from other methods; researcher influence on data not stated; research philosophy not stated; description of data analysis lacks detail
[70]	To explore and describe nurse practitioners' experiences and perceptions of interprofessional collaboration with MPs in PHC	Naturalist inquiry<br><br>Qualitative descriptive study design	6 NPs<br><br>Purposive and snowball sampling	1 Family Health Team, 1 PHC Network, 2 Community clinics, 1 Community Health Centre; Ontario, Canada	- semi-structured interviews,<br>- content analysis	Seven themes:<br>- quality of communication,<br>- complementary vision,<br>- physician remuneration methods,<br>- establishing and maintaining relationships,<br>- investing time and energy,<br>- nurse practitioner competency and expertise<br>- mutual trust and respect	Strengths: Well described study, participant voices are well presented, findings are credible<br><br>Limitations: Researcher background stated but influence on data not discussed
[57]	To examine the	Qualitative	10 MPs	Family practice,	Semi-structured	- MPs concerned about independent NP practice.	Limitations: Lack of participant citations

	perceptions of family MPs toward NPs and physician assistants	study	(residents)<br><br>Convenience sample of MPs with random selection of participants	Southeast US	interviews	- MPs made positive comments about NPs, but the approval was generally based on the NP's adherence to guidelines<br>- MPs feel more comfortable with NPs in traditional roles.<br>- MPs misinformation about NP role and qualification.<br>- Diagnostic skills of NPs are limited (perceived by MPs)<br>- NPs can alleviate MPs workload<br>- NPs are cost-effective<br>- Positive attitude towards NPs from MPs who had experience in working with them.	to illustrate findings, researcher influence on data not stated; research philosophy not stated<br><br>EXCLUDED FOR ANALYSIS
[17]	To examine the phenomenon of MP valuing of NPs in rural PHC clinics	Naturalist Inquiry<br><br>Descriptive exploratory design	10 MPs<br><br>Convenience sampling	Rural PHC clinics, Central/southern Missouri, US	Semi-structured interviews	3 overarching themes: NPs value to the MP, to the practice, to the patient<br>- Differing perceptions of NPs and MPs about collaboration<br>- Lack of reciprocity<br>- MPs conceptualisation of collaboration is not conform to the ideal described in literature.	Strengths: participant voices are well presented, findings are credible, researcher background and influence on data stated<br><br>Limitations: Short interviews (10-30 min) may lack in-depths data, not very well written
[79]	To elicit data about MPs' knowledge and ideas about working with NPs	Qualitative study	8 resident MPs, 3 faculty MPs<br><br>Convenience sampling	Family Medicine Residency; Manitoba, Canada	3 focus group discussions	- Concern voiced by MPs towards collaboration<br>- Advantages seen by MPs to work in collaboration<br>- Barriers: NP education not equivalent to MP education, so NPs is not seen as equal partners; lack of understanding of skills of a NP	Strengths: researcher background stated<br><br>Limitations: Poor reporting, research philosophy not stated, description of data analysis lacks detail
[69]	To investigate and describe the experiences of advanced practice nurses (APN) and of their supervising MP, regarding the role and scope of practice	Qualitative study based in Anthropology	4 APNs (similar to NPs),<br>5 MPs<br><br>Purposeful sampling	PHC centres, Sweden	- Interviews with APNs<br>- Focus groups with MPs	Four themes were developed: Confidence and trust, the positioning of old and new roles (establishing role clarity), demarcation, expectations and experience of the NP as a resource	Strengths: participants well represented, findings credible<br><br>Limitations: researcher background and influence on data not stated
[63]	To investigate how the working patterns of PHC teams have been altered as a result of the introduction of NPs, the ways in which NPs' skills are integrated into the team and perceptions of the NP role	Qualitative study	Interviewed:<br>4 NPs<br>3 GPs<br>Focus groups:<br>3 GPs, 3 NPs<br>6 practice nurses,<br>3 practice managers<br>3 receptionists<br><br>Purposeful sampling	PHC practices, Northern Ireland	- Focus groups<br>- semi-structured interviews	- Barriers: MPs have more time for complex cases (which by some has been experienced as stressful and some were concerned about becoming de-skilled in some areas), lack of understanding of the NP role, lack of clarity about legal situation for NPs<br>- Facilitators: Respect from colleagues, support from MPs who had previously known the NPs, knowing your own limitations (perceived by NPs), official recognition of the NP role	Strengths: participants well represented, findings credible<br><br>Limitations: research philosophy and researcher background not stated
[64]	To explore, how health professionals perceive the role of NPs in PHC	Qualitative study, (Grounded theory)	10 GPs,<br>8 NPs,<br>1 practice nurse<br>2 managers<br><br>Purposeful sampling	5 PHC Centres; Southampton City, UK	Semi-structured interviews	Barriers to NP role: organisational factors, training and prescribing issues, lack of a professional register, and cultural issues including tensions, boundaries and responsibility.	Strengths: participant voices are well presented, findings are credible<br><br>Limitations: Authors claims to use grounded theory but no theory has been developed, rather descriptive presentation of findings; researcher influence on data not discussed
[65]	To obtain the views of members of the PHC team about the NP role and to explore how this was perceived to	Qualitative study	9 GPs<br>(other staff, total of 27)<br><br>Convenience sampling	4 GP practice, North-West England	Semi-structured interviews in groups and with individuals	- MPs unclear about NP role<br>- MPs experienced release of consultation time<br>- MPs concerned about ultimate responsibility<br>- MPs ambivalent about cost effectiveness of NP	Strengths: participant voices are well presented, findings are credible<br><br>Limitations: Data from 2000, research philosophy, researcher background and influence on data not stated

	impact on them, the practice and patient care.		(practices), Purposive sampling (staff)				
[67]	To explore the role and practice of NPs in general practice.	Qualitative study	4 NPs<br>4 GPs<br>(4 receptionists, 24 patients)<br><br>Convenience/ snowball sampling (practices), Purposive sampling (staff)	4 general practices; South-East England	Semi-structured in depths interviews	- Barriers: MPs concerned about legal responsibility<br>- Facilitators: support from MPs, higher level of NP autonomy<br>- Reduction of MP workload through NP<br>- MP defines work that is delegated to the NP	Strengths: participant voices are well presented, findings are credible<br><br>Limitations: Ethics approval not reported, Research philosophy not reported, Data analysis method unclear, researcher background and influence on data not stated.
[66]	To explore views of GPs regarding their attitudes towards NP role	Qualitative study	25 GPs<br><br>Purposeful sampling	4 GP practices, Yorkshire, UK	Focus groups	- Themes: NPs concerned about their status including job and financial security, about nursing capabilities including training and scope of responsibility, and about structural and organisational barriers	Strengths: Participants well presented, results are credible, large sample size suggests generalisability to similar setting.<br><br>Limitations: Ethics approval not reported, research philosophy not reported, researcher background and influence on data not stated.
**Survey studies**							
[14]	To test a theoretical model linking NP's perceptions of workplace empowerment, collaboration with MPs and managers, and job strain.	Mailed survey	54 PHC NPs (and 63 acute care NPs, not included in this review)<br><br>Convenience sample of registered nurses who indicated working as NP	Ontario, Canada	- Kanter's structural theory of power in organizations<br>- Survey including 'Conditions of work effectiveness questionnaire', 'Collaborative behaviour scale', 'Job content questionnaire'	- NP workplace empowerment positively related to collaboration with MPs (r=.442, p=.0001)<br>- NP's perceptions of job strain negatively related to collaboration with MPs (r=-.362, p=.004)	Strengths: Validated tools<br>Good response rate<br><br>Limitations: Limited generalisability due to convenience sample.
[77]	To evaluate factors associated with MPs' attitudes toward NPs providing PHC.	Mailed survey	259 PHC MPs<br><br>Random selection of PHC MPs of list with all MPs in Iowa	non-institutional-based PHC sites;<br>Iowa, US	Survey (11-item questionnaire with 5 point Likert scale)	- MPs had more favourable attitudes towards NPs when they had previous experience working with NPs providing PHC (P = .01)<br>- MPs were more likely to have had experience with an NP providing PHC if they were in pediatrics or obstetrics-gynecology (78.3% and 70.0%, respectively; P <.001), had been in practice for fewer than 20 years (P = .045), or were in practices with 5 or more MPs.<br>- Age, sex, years in practice, and practice size, were not significantly related to MP attitude.	Strengths: Validated tool; Random sampling<br><br>Limitations: Low response rate (42%); Data from 1994
Bergeson et al. 1997 *	To assess MPs' awareness of and attitudes toward the use of physician assistants and NPs	Mailed survey and follow-up interviews	277 family MPs<br><br>Convenience sampling	Non-urban towns in Minnesota, US	Self-developed mixed methods questionnaire with Likert-Scales and free text fields.<br>Telephone interviews with 22 MPs	- 66.2% of MPs who had previously worked with NPs indicated their experience as positive, 21.5% as somewhat positive, 7.3% as neutral, 4.6% as somewhat negative and 0.5% as negative.<br>- (other results were not reported separately for NPs and physician assistants)	Strengths: Data validation through follow-up interviews<br><br>Limitations: Low response rate (46.2%); no psychometric properties reported for questionnaire, data analysis process of qualitative interview data unclear
[76]	To investigate GP's perceptions of the NP role	Mailed survey	225 GPs	Lincolnshire and Sheffield, UK	- Self developed questionnaire with open and closed questions<br>- descriptive and inferential statistics<br>- content analysis	- More acceptance of NPs by MPs who employ NP<br>- Different opinions between MPs who employ and who do not employ NPs<br>- Reason to employ NPs: increased patient choice, reduced workload, more cost effective use of resources, MP shortage, reduced waiting times.	Strengths: Large sample size<br><br>Limitations: Low response rate (33%); no psychometric properties of questionnaire reported; correlational analysis not undertaken for all results; findings from

							qualitative data not presented
[13]	To identify the barriers and facilitators associated with the implementation of the NP role and the NPs' job satisfaction	Mailed survey	28 NPs<br><br>Convenience sampling	Public Health Units;<br>Ontario, Canada	Questionnaire with 6-point Likert scale based on questionnaire used in a previous study (included the ranking of barriers/facilitators)	- Facilitators: trust shown by the MP in making shared decisions, respect shown by the MP, personality and philosophy of the MPs<br>- Barriers: most frequent: unwillingness of specialists to accept referrals from the NP, MP lack of understanding of the NP role, personality and philosophy of the MPs<br>- NPs generally "satisfied" with collaborative relationship with the MP<br>- NP work satisfaction positively correlated with satisfaction with their collaborative relationship with the MP (r = 0.59, p<0.01).<br>- NP work satisfaction negatively correlated with the number of barriers present in their relationships with the MP (r = -0.46, p<0.05).	Strengths: Very high response rate (95%), generalisable within NP population<br><br>Limitations: Sample size too small to detect significant differences; no psychometric properties of questionnaire reported
[68]	To describe NPs' and MDs' perceptions of the role of NPs, the degree of collegiality between professions, and NPs' feeling of acceptance.	Mailed survey<br><br>Part of a mixed methods survey [75].<br>This paper focuses on 4 open-ended questions	74 NPs,<br>79 MPs<br><br>Convenience sampling	7 Veterans affairs outpatient clinics;<br>Michigan, Indiana, Illinois, Ohio, US	Closed- and open-ended questions plus several Likert-type questions	Three themes identified: Roles of the NP in PHC, workload reduction of MPs, clinical competence or independence of NPs.<br><br>Results from quantitative data report attitudes of NPs and MPs towards collaboration	Strengths: Good overall response rate (61.4%), data validation through mixed-methods questionnaire<br><br>Limitations: Participant selection process unclear, low response rate for MPs (49%), no psychometric properties of questionnaire reported
[75]	To examine the perceptions of NPs and MPs regarding NPs' roles as PHC providers	Mailed survey<br><br>Part of a mixed methods survey [68].<br>This paper reports quantitative data	74 NPs,<br>79 MPs<br><br>Convenience sampling	7 Veterans affairs outpatient clinics;<br>Michigan, Indiana, Illinois, Ohio, US	Closed- and open-ended questions plus several Likert-type questions	- NPs and MPs agreed on NP independence of care for chronic patients, but not for acute patients.<br>- NPs were significantly more likely than MPs to indicate they independently conducted assessments, planned care, added or changed medications, and performed other unspecified activities for acute patients (p < 0.01)<br>- NPs were more likely to care for patients with less comorbidity while MPs cared for patient with more comorbidity.	Strengths: Good overall response rate (61.4%), Confounding factors included in analysis<br><br>Limitations: Data from 2004, low response rate for MPs (49%), no psychometric properties of questionnaire reported
[71]	To explore the attitudes and beliefs of pediatric NPs and pediatricians concerning collaborative practice relationships;<br>and to explore the themes that emerged to establish a definition of collaborative practice between NPs and pediatricians	Mailed survey	24 pediatric NP and pediatrician dyads<br><br>Random sampling from list of NPs	Paediatric PHC practices<br>US	- Mixed methods questionnaire with open ended questions and Likert scale rating<br>- Collazzi's phenomenological methodology used for data analysis	- Definition of collaboration: (4 themes): Working together/collegial relationship, consultation, share philosophy/goals, complimentary practice styles/comfort level<br>- Facilitators: Trust and mutual respect, communication, shared practice, competence (from NP data), similar vision (from MP data)<br>- Barriers: Lack of respect, territorial/control issues, undesirable attitude/behavior of MPs, lack of competence (from NP data); Control/inflexible, NP competence in clinical practice, ineffective communication (from MP data)<br>- Differing understandings of supervision and independence<br>- Trust, clinical competence, knowing when to seek consultation were rated high as important characteristics of collaboration by NPs and MPs	Strengths: Random selection of participants, rigorous analysis method, data validation through mixed-methods questionnaire<br><br>Limitations: Low response rate (17.3%); not all themes are supported with quotes.
[74]	To identify the perceptions of NPs, MPs, pharmacists and nurses towards safety climate, communication and collaboration in PHC.	Survey	12 NPs,<br>39 MPs<br>(46 nurses, 10 pharmacists)<br><br>Convenience sample	4 military ambulatory care clinics;<br>Midwestern US	Safety Attitudes Questionnaire (77items), Likert-scales; adapted from the 'Flight Management Attitudes Questionnaire'	- 90.9% of NPs rated MPs as high/very high on collaboration or communication<br>- 82.8% of MPs rated NPs as high/very high on collaboration or communication	Strengths: Validated tool, good response rate (65%)<br><br>Limitations: Sample size too small to detect significant differences

[62]	To compare perceptions of NPs and MPs about NP role	Mailed survey	28 family NPs, 37 family MPs<br><br>Random sampling from list of participants	Air Force installations, US	Self-developed questionnaire with Likert scales to rank the appropriateness of tasks for NPs; and questions for perceived barriers to NP deployment	- NPs perceived that they could independently treat 66% of 65 symptom/illness categories. The MPs perceived that NPs could only treat 29% of those categories.<br>- Differences between NPs and MPs in a number of disease/illness areas for which NPs would need MP supervision.<br>- 38% of MPs thought that NPs require supervision of an MP	Strengths: Good response rate (81%), validated tool, random sampling, results likely to be generalisable<br><br>Limitations: Randomisation process not clearly described
[81]	To explore perceptions of GPs in regarding the NP role, identifying their knowledge of and perceived problems with that role, and their experience of nurses in advanced practice.	Mailed survey	50 GPs<br><br>Convenience sampling	GPs in Northland District, New Zealand	Questionnaire, 5-point Likert scales, adapted from the 'Survey of General Practice Physicians' Opinion Concering the Family Nurse Practitioner'	- 64% of MPs said they would be willing to employ an NP; and 86% indicated a willingness to work in collaboration with an NP<br>- MPs reluctant to NP authority for prescribing, ordering tests and undertaking physical assessment.<br>- Uncertainty about NP role and competence	Strengths: Results are well presented<br><br>Limitations; Limited generalizability due to low response rate (46.3%) and convenience sampling, no psychometric properties reported
[80]	To understand the acceptability for a model of chronic disease management, in which PHC patients see NPs for structured visits	Mailed survey	95 NPs,<br>77 MPs<br><br>Random sampling	Metropolitan PHC practices, Philadelphia, Pennsylvania, US	Self-developed questionnaire with 4 point Likert scales	- Most MPs and NPs believed that the proposed model of care would improve the control of chronic illnesses.<br>- The logistic regression modeling revealed that NPs were 4.2 times more likel<br>y to support the model of care than were MPs ($P \leq .001$; confidence interval [CI], 2.1-8.3).	Strengths: random sampling, validated outcome measure, potential confounders considered<br><br>Limitations: Results may not be generalisable due to low response rate (53%)
[78]	To analyse how MP characteristics and close working relationships influence MPs' attitudes toward NPs	Online and mailed survey	463 MPs<br><br>Convenience sampling	Mississippi, US	Questionnaire developed by Aquilino et al. [85] with Likert scales<br>(part of omnibus survey)	- GPs, MPs in public sector and MPs in larger practices are more likely to work in practices that also include NPs<br>- MPs working with NPs are somewhat younger than those who do not.<br>- MPs who practice alongside NPs and who have been in practice longer have the most positive attitudes toward NPs.<br>- MPs had more favourable attitudes towards NPs when they had previous experience working with NPs	Strengths: validated tool, confounding factors considered, large sample size.<br><br>Limitations: Low response rate (23.3%) and convenience sampling limits generalisability
**Mixed-Methods studies**							
[58]	To examine the development of collaborative relationships between family MPs and other team members	Qualitative evaluation of RCT<br><br>Part of a mixed methods study	8 family MPs,<br>3 NPs<br>1 pharmacist	Family Practice, Ontario, Canada	- Collaboration Care Provider Survey (5, 12, 18 months)<br>- focus groups<br>- in-depths interviews<br>- case study (each provider)<br>- daily logs by NP/pharmacist	Barriers: Lack of role clarity, geographic separation, MPs concerned about legal responsibility of shared care.<br>- Facilitators: Regular meetings, clarifying responsibilities, prior experience of working with NPs, phone messaging system to facilitate contact among each other.<br>- approx. 6 months needed to establish an understanding of the areas of competency, scope of practice, individual strengths<br>- Collaboration as the ideal practice was not always attained.	Strengths: Validated tool for quantitative measures, data validation through mixed-methods approach<br><br>Limitations: NP/MP sampling process not described, researcher background and influence on data not stated
[19]	To develop, implement and evaluate an intervention to support NP/family MP structured collaborative practice, including the evaluation of satisfaction levels,	Mixed-methods study with quasi-experimental design<br><br>Project-related publications [59,60]	5 NPs<br>13 MPs<br><br>Purposeful sampling	4 PHC practices,<br>(2 control, 2 intervention sites)<br>Ontario, Canada,	- Surveys and interviews of NPs/MPs, patients and key informants<br>- patient encounter forms	- Barriers: Medico-legal concerns by MPs, lack of knowledge about NP role, practice structural and ideological differences (health promotion), lack of financial support<br>- Facilitators: bi-directional consultation and referrals, working side-by-side at the same clinic, previous experience of working with NPs, clarification of values/ expectations about collaboration through discussion, use of technologies to facilitate collaboration across distance<br>- NP and MPs in intervention sites had higher level of collaboration and higher satisfaction with collaboration post	Strengths: Comprehensive evaluation of NP-MP collaboration<br><br>Limitations: Self-reported data on referrals from NPs/MPs, questionable to measure shared care based on referral patterns, small sample size limits generalisability

	change of attitudes towards collaboration over the course of project and identification of barriers and facilitators to collaboration					intervention.	
**Cross-sectional study**							
[59]	To determine which services are provided to patients by NPs and MPs and to determine the degree of collaboration/ shared care.	Mixed methods cross sectional study<br><br>(this paper reports quant results of a larger mixed methods study) [19,60]	5 NPs<br>13 MPs<br><br>Purposeful sampling	4 PHC practices Ontario, Canada	- Encounter forms filled out by NPs and MPs (400 patients encounters)<br>- Referral mechanisms used to measure shared care/collaboration<br>(- Patient interviews, not reported in this paper)	- Comparison of task of NPs and MPs: NPs similarly involved in curative services than MPs, NPs less involved in rehabilitation, more involved in disease prevention.<br>- 16% of NP referrals were to MPs; 2% of referrals by MPs were to NPs (unidirectional referrals)<br>- Underutilisation of NP skills	Strengths: Comprehensive evaluation of NP-MP collaboration<br><br>Limitations: Self-reported data on referrals from NPs/MPs, questionable to measure shared care based on referral patterns, small sample size limits generalisability
PHC = Primary Health Care, NP = Nurse Practitioner, MP = Medical Practitioner, GP = General Practitioner, APN = Advanced Practice Nurses, US = United States of America, UK = United Kingdom							

<u>Reference, not used in review</u>

* Bergeson J, Cash R, Boulger J, Bergeron D: The attitudes of rural Minnesota family physicians toward nurse practitioners and physician assistants. J Rural Health 1997, 13(3):196-205.
