# Supplementary material for: Views and experiences of nurse practitioners and medical practitioners with collaborative practice in primary health care – an integrative review
Source: BMC Fam Pract. 2013 Sep 5;14:132. doi: 10.1186/1471-2296-14-132 (PMC3846155; doi:10.1186/1471-2296-14-132)
Supplement: Additional file 3 — Quality appraisal, Table that outlines the quality appraisal of included studies. [file 1471-2296-14-132-S3.pdf]

**Quality appraisal of studies**

| **Qualitative studies** | [61] | [60] | [72] | [73] | [70] | [57] | [17] | [79] | [69] | [63] | [64] | [65] | [67] | [66] |
|---|---|---|---|---|---|---|---|---|---|---|---|---|---|---|
| Philosophy congruent with methodology | U | U | U | U | + | U | + | U | + | U | U | - | - | U |
| Methodology congruent with aim | + | + | + | + | + | + | + | U | + | + | + | + | + | + |
| Methodology congruent with data collection methods | U | + | + | + | + | + | + | + | + | + | + | + | + | + |
| Methodology congruent with analysis | - | + | + | U | + | + | + | U | + | + | + | + | U | + |
| Methodology congruent with interpretation of results | - | + | + | + | + | + | + | U | + | + | - | + | + | + |
| Researcher background stated | - | - | - | - | + | - | + | + | - | - | - | - | - | - |
| Influence of researcher stated | - | - | - | - | - | - | + | + | - | U | - | - | - | - |
| Participant voices adequately represented | U | + | + | + | + | U | + | U | + | + | + | + | + | + |
| Ethical approval | U | + | + | + | + | + | + | U | + | + | + | - | - | U |
| Conclusions derived from data | - | + | + | + | + | + | + | + | + | + | + | + | + | + |
| **Survey studies** | [14] | [77] | * | [76] | [13] | [68] | [75] | [71] | [74] | [62] | [81] | [80] | [78] | |
| Clear aim addressed | + | + | + | + | + | + | + | + | + | + | + | + | + | |
| Design appropriate for research question | + | + | + | + | + | + | + | + | + | + | + | + | + | |
| Clear selection process | + | + | + | + | + | - | + | + | + | + | + | + | + | |
| Potential for selection bias | + | - | + | + | - | U | + | - | + | - | + | - | + | |
| Subjects are representative | - | - | - | - | + | - | - | - | - | + | - | - | - | |
| Power analysis included | - | - | NA | - | - | NA | - | - | - | - | NA | - | - | |
| Response rate in % (satisfactory if ≥ 60%)** | 68.8 | 42 | 46.2 | 33 | 96.5 | 61.4 | 61.4 | 17.3 | 65 | 81% | 46.3 | 53 | 23.3 | |
| Valid and reliable measurements | + | + | U | U | U | U | U | U | + | + | U | + | + | |
| Statistical significance assessed | + | + | NA | + | + | NA | + | + | + | U | NA | + | + | |
| Confidence intervals given | - | - | - | - | - | NA | - | - | - | - | NA | - | - | |
| Unaccounted confounding factors present | + | + | U | + | + | NA | - | + | + | + | NA | - | - | |
| **Mixed-methods studies** | [58] | [19] | | | | | | | | | | | | |
| Qualitative objective present | + | + | | | | | | | | | | | | |
| Design/methods appropriate for research question | + | + | | | | | | | | | | | | |
| Context described | + | + | | | | | | | | | | | | |
| Participants described & sample justified | - | + | | | | | | | | | | | | |
| Qual data collection & analysis described | + | + | | | | | | | | | | | | |
| Researcher's reflexivity described | - | - | | | | | | | | | | | | |
| Quant sampling & sample appropriate | - | - | | | | | | | | | | | | |
| Justification of measurements (validity) | + | + | | | | | | | | | | | | |
| Confounding variables controlled | - | - | | | | | | | | | | | | |
| Mixed-methods design justified by authors | - | 1 | | | | | | | | | | | | |
| Combination of qual & quant data collection & analysis | + | + | | | | | | | | | | | | |
| Integration qual & quant results | + | - | | | | | | | | | | | | |
| **Descriptive cross-sectional study** | [59] | | | | | | | | | | | | | |
| Clear aim addressed | + | | | | | | | | | | | | | |
| Methods appropriate for research question | + | | | | | | | | | | | | | |
| Sample representative | - | | | | | | | | | | | | | |
| Measurements accurate and valid | U | | | | | | | | | | | | | |
| Data collection method appropriate | + | | | | | | | | | | | | | |
| Participant number large enough | U | | | | | | | | | | | | | |
| Results correct presented | + | | | | | | | | | | | | | |
| Analysis correct | - | | | | | | | | | | | | | |
| Findings clearly stated | + | | | | | | | | | | | | | |

+ = yes; - = no; U = unclear; NA = Criterion not applicable because of descriptive design;
* Bergeson J, Cash R, Boulger J, Bergeron D: The attitudes of rural Minnesota family physicians toward nurse practitioners and physician assistants. J Rural Health 1997, 13(3):196-205.
** 60% was chosen based on recommendations in the literature
